# Chloroplast population genetics reveals low levels of genetic variation and conformation to the central–marginal hypothesis in *Taxus wallichiana* var. *mairei*, an endangered conifer endemic to China

**DOI:** 10.1002/ece3.5703

**Published:** 2019-09-27

**Authors:** Li Liu, Zhen Wang, Lijie Huang, Ting Wang, Yingjuan Su

**Affiliations:** ^1^ School of Life Sciences Sun Yat‐sen University Guangzhou China; ^2^ College of Life Sciences Nanjing Agricultural University Nanjing China; ^3^ College of Life Sciences South China Agricultural University Guangzhou China; ^4^ Research Institute of Sun Yat‐sen University Shenzhen China

**Keywords:** central–marginal hypothesis, climatic variable, cpSSRs, plastid genetic variation, *Taxus wallichiana* var*. mairei*

## Abstract

The central–marginal hypothesis predicts that geographically peripheral populations should exhibit reduced genetic diversity and increased genetic differentiation than central populations due to smaller effective population size and stronger geographical isolation. We evaluated these predictions in the endangered conifer *Taxus wallichiana* var*. mairei*. Eight plastid simple sequence repeats (cpSSRs) were used to investigate plastid genetic variation in 22 populations of *Taxus wallichiana* var. *mairei*, encompassing nearly its entire distribution range. Low levels of plastid genetic variation and differentiation were detected in the populations, and the findings were attributed to low mutation rates, small population sizes, habitat fragmentation and isolation, and effective pollen or seed dispersal. Hunan and Hubei were identified as major refugia based on the number of private haplotypes and species distribution modeling. Trends in plastid genetic diversity and genetic differentiation from central to peripheral populations supported the predictions of the central–marginal hypothesis. In scenarios wherein the future climate becomes warmer, we predict that some peripheral populations will disappear and southern and southeastern regions will become significantly less habitable. Factors that include the levels of precipitation during the driest month, annual precipitation level, and annual temperature range will be decisive in shaping the future distribution of these populations. This study provides a theoretical basis for the conservation of *T. wallichiana* var. *mairei*.

## Graphical abstract

We detected low levels of chloroplast genetic variation and differentiation in the populations of *Taxus wallichiana* var. *mairei*. We identified Hunan and Hubei as major refugia. Our results supported the predictions of the central–marginal hypothesis. We clarified factors that will be decisive in shaping the future distribution of the populations.

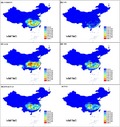

## 1 INTRODUCTION

Knowledge of population genetic patterns across a species' geographical range is of crucial importance to predict its responses to climate change (MacArthur, 1972; Trumbo et al., 2016). As one of the major evolutionary hypotheses, the central–marginal hypothesis (CMH) makes specific predictions in this regard (Eckert, Samis, & Lougheed, 2008; Sexton, McIntyre, Angert, & Rice, 2009). It predicts that geographically peripheral populations should exhibit reduced genetic diversity and increased genetic differentiation than central populations due to smaller effective population size and stronger geographical isolation (Eckert et al., 2008; Trumbo et al., 2016). If this is true, peripheral populations will have limited evolutionary potential to adapt to habitat conditions beyond current range limits. However, ecological processes such as phenotypic plasticity and migration and their interplay with evolutionary changes may play equally important roles in the persistence of species in changing environments (Aitken, Yeaman, Holliday, Wang, & Curtis‐McLane, 2008; Anderson, Panetta, & Mitchell‐Olds, 2012).

It has been noted that climate change can affect the fate of peripheral populations by the following mechanisms: (a) occurrence of significant evolutionary changes; (b) an increase in the risk of extinction; (c) peripheral populations becoming the leading edge of migrations; (d) genetic novelty being created and perhaps reinforcing standing genetic variation; and (e) fluctuations in the extent and scale of local adaptations being generated (Fady et al., 2016; Marschalek & Berres, 2014). Pfeifer et al. (2009) pointed out that conservation of peripheral populations is potentially valuable. In this context, a test of the CMH with rare species is relevant to forecast range shifts under climate change as well as to evaluate the conservation concern of peripheral populations.

Empirical tests of the CMH have found support in a range of plant taxa (Hampe & Petit, 2005; Myking, Vakkari, & Skrøppa, 2009), but not others (Dixon, Herlihy, & Busch, 2013; Garner, Pearman, & Angelone, 2004; Munwes et al., 2010). Theoretically, it is quite challenging to disentangle the causes of a central–marginal structure of genetic diversity, because factors such as biological characters, persistent historical influences, effects of current demographic and ecological variables, population sampling strategies, and molecular markers applied for analysis may all exert impacts (Loveless & Hamrick, 1984; Schiemann, Tyler, & Widén, 2000; Wagner, Durka, & Hensen, 2011). In this study, we tested the genetic predictions of the CMH by using plastid simple sequence repeats (cpSSRs) to assess populations of the endangered conifer *Taxus wallichiana* var*. mairei* (Taxaceae).

*Taxus wallichiana* var*. mairei*, previously classified as *Taxus chinensis* var*. mairei,* is a well‐known tertiary relict yew endemic to China (Fu, Li, & Mill, 1999; Zheng & Fu, 1978). Its populations are naturally scattered, with a typical central–marginal distribution pattern (Su, Wang, & Ouyang, 2009; Wu & Wen, 2017; Zhang & Zhou, 2013). The size of *T. wallichiana* var*. mairei* populations has declined significantly over the past few decades because of overexploitation, slow growth, and long seed dormancy requirements (Zhang, Liao, Zhong, & Chen, 2000). Currently, the plant is listed as a first‐class protected species in China (Bao & Chen, 1998).

The genetic diversity of *T. wallichiana* var*. mairei* has been investigated using nuclear inter simple sequence repeat (ISSR) and nuclear simple sequence repeat (nSSR) markers (Zhang & Zhou, 2013; Zhang, Gao, Möller, & Li, 2009). ISSRs revealed that *T. wallichiana* var*. mairei* has high genetic diversity and genetic differentiation (Zhang et al., 2009), whereas nSSRs uncovered moderate genetic diversity and low genetic differentiation (Zhang & Zhou, 2013). Nevertheless, the level of genetic variation within the plastid genome of *T. wallichiana* var*. mairei* remains unclear (Zhang et al., 2014). CpSSRs are particularly useful plant molecular markers. They are haploid, nonrecombinant, inherited from only one parent (Ebert & Peakall, 2009), and highly polymorphic (Provan, Powell, & Hollingsworth, 2001; Provan, Soranzo, Wilson, Goldstein, & Powell, 1999). Importantly, plastid DNA can provide information on historical bottlenecks, founder effects, and genetic drift because it preserves ancient genetic patterns and has a smaller effective population size than the nuclear genome (Martins et al., 2013; Provan et al., 2001). Such information is complementary and comparable with that obtained from nuclear DNA (Petit, El Mousadik, & Pons, 1998; Powell, Machray, & Provan, 1996; Provan et al., 2001).

Global warming may alter environments suitable for many species of conifer, forcing them to migrate north (Ettinger & HilleRisLambers, 2017; Quiroga, Premoli, & Kitzberger, 2018; Wang, Wang, Xia, & Su, 2016). Climate is known as the most important factor shaping the natural distribution of *T. wallichiana* var. *mairei*. As the plant prefers moist and shady habitats, its widespread distribution particularly makes it sensitive to climatic changes (Wu & Wen, 2017). Therefore, understanding the distribution of *T. wallichiana* var*. mairei* in terms of past, present, and future climate conditions would provide a good basis for conservation strategies. It would be valuable to identify the climatic variables that make the most significant impact on its distribution.

In this study, we used cpSSRs to investigate *T. wallichiana var. mairei* populations from across China. Our goals were (a) to reveal the level of plastid genetic diversity and genetic differentiation; (b) to test the central–marginal hypothesis by examining the plastid genetic variation between central and marginal populations; and (c) to define the ecologically suitable area for these populations and determine the decisive climatic factors limiting their distribution.

## 2 MATERIALS AND METHODS

### 2.1 Study species and study range

*Taxus wallichiana var. mairei* (Taxaceae) is a slow‐growing tree that varies in height from 2.5 to 20 m (Fu et al., 1999). Its needle leaves with white stomatal bands are arranged in a spiral along the stem, and brightly colored arils that are attractive to birds facilitate seed dispersal. As a dioecious and wind‐pollinated coniferous species, *T. wallichiana* var. *mairei* occurs as part of the understory of mixed forests located in mountains and valleys at an altitude of 1,000–1,500 m (Fu et al., 1999; Zheng & Fu, 1978). The plant is rich in the anticancer agent Taxol and can also be used to make high‐quality redwood furniture (Fan, Tang, & Shu, 1996).

*Taxus wallichiana* var. *mairei* is typically found in the subtropical to warm temperate zone in China. It is widely distributed across regions and provinces, including Gansu, Shanxi, Sichuan, Yunnan, Guizhou, Hubei, Hunan, Zhejiang, Guangxi, Anhui, and Taiwan, with the Yangtze River basin and Nanling Mountains at the center of its range (Wu & Wen, 2017; Yang, Dick, Yao, & Huang, 2016).

### 2.2 Plant materials

**Figure 1:**
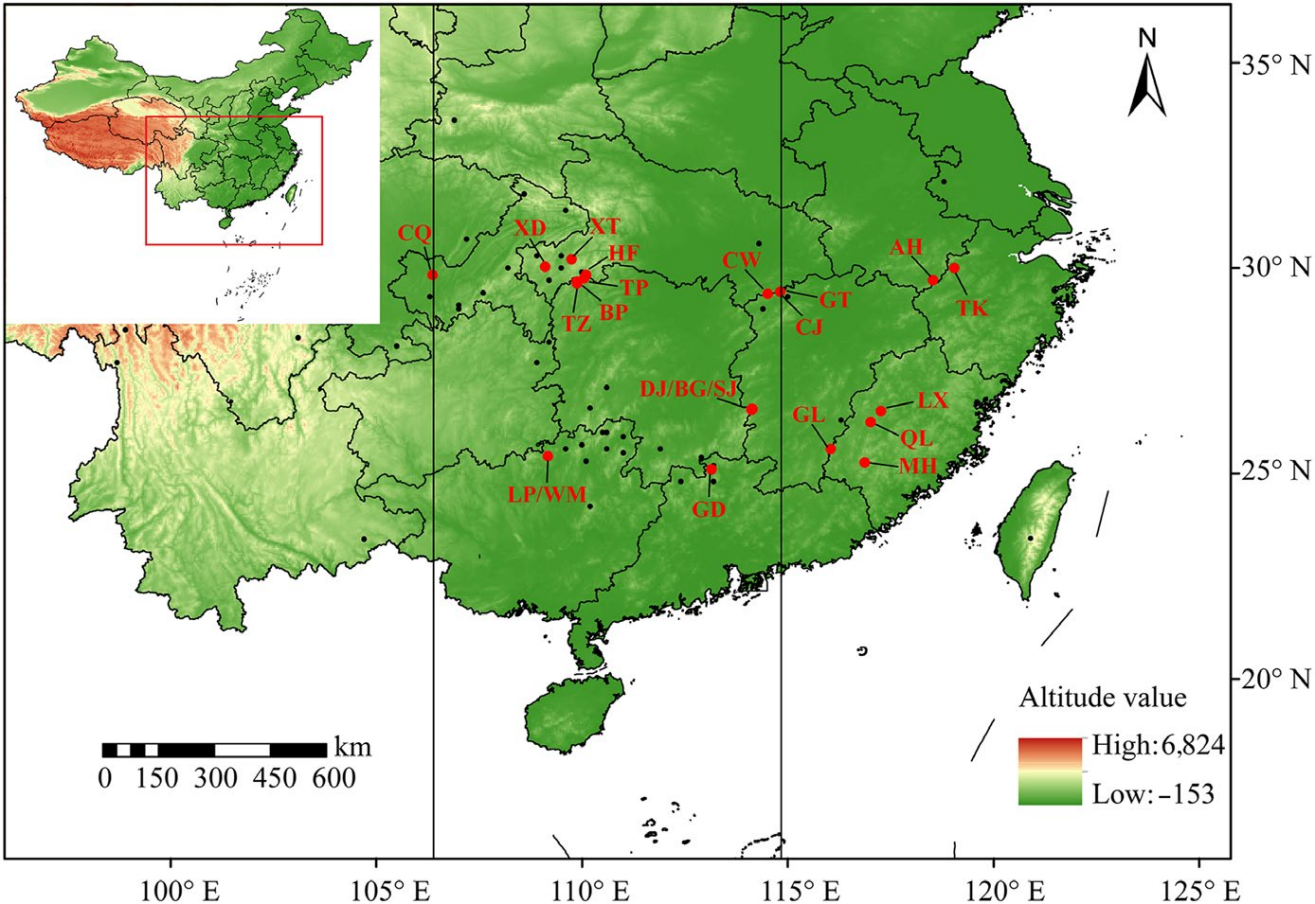
Sampling locations and geographical distribution of the 22 *Taxus wallichiana* var. *mairei* populations (Red dots). See Table 1 for locality abbreviations. The central and peripheral populations were separated by two perpendicular lines. The small black dots show the recorded locations of *T. wallichiana* var. *mairei* populations. The color‐scale key indicates the altitude value in meters

A total of 339 individuals were sampled from 22 populations, which covered almost the entire range of *T. wallichiana* var*. mairei's* distribution across China (Figure 1, Table 1). For each population, 4–20 individuals, located at least 20 m apart, were randomly selected. Fresh young leaves were dried immediately in silica gel for DNA extraction.

**Table 1:** The location and genetic diversity of *Taxus wallichiana* var. *mairei* populations as determined by cpSSR analysis

Populations	Group	Sample sizes	Locations	Longitude (E)	Latitude (N)	*N*_p_	PPB (%)	*N*_a_	*N*_e_	*h*	*I*	*H*_d_
TP	C	10	Tianping Mount, Hunan	110°01′	29°23′	4	50.00	1.750	1.3097	0.1750	0.3018	0.600
TZ	C	20	Taiziping, Hunan	109°42′	29°11′	6	75.00	2.125	1.3803	0.2437	0.4114	0.600
BP	C	20	Bapi, Hunan	109°42′	29°10′	3	37.50	1.750	1.3707	0.1400	0.2560	0.133
HF	C	20	Hefeng, Hubei	110°19′	29°58′	4	50.00	1.875	1.3457	0.1875	0.3222	0.500
XT	C	20	Xintang, Hubei	109°46′	30°12′	3	37.50	1.625	1.4162	0.1569	0.2679	0.450
XD	C	20	Xingdou Mount, Hubei	108°55′	30°12′	4	50.00	1.875	1.2418	0.1500	0.2688	0.450
GT	C	11	Gaotai, Hubei	114°35′	29°47′	4	50.00	1.625	1.1889	0.1302	0.2244	0.545
CJ	C	13	Chengjia, Hubei	114°35′	29°47′	3	37.50	1.500	1.1409	0.9760	0.1735	0.462
CW	C	20	Chuangwang, Hubei	114°35′	29°47′	4	50.00	1.500	1.1287	0.0931	0.1606	0.250
AH	M	11	Changgai, Anhui	118°24′	30°03′	0	0.00	1.000	1.0000	0.0000	0.0000	0.091
GL	M	12	Guilong Mount, Fujian	116°44′	25°57′	3	37.50	1.500	1.2251	0.1302	0.2109	0.417
MH	M	12	Meihua Mount, Fujian	117°14′	25°23′	2	25.00	1.250	1.0451	0.0382	0.7170	0.167
LX	M	20	Longxi Mount, Fujian	117°34′	27°12′	2	25.00	1.375	1.0982	0.0625	0.1161	0.200
QL	M	4	Qingling, Fujian	117°09′	26°46′	0	0.00	1.000	1.0000	0.0000	0.0000	0.250
TK	M	20	Duankou, Zhejiang	119°18′	29°51′	4	50.00	1.625	1.1588	0.1181	0.2086	0.350
DJ	C	14	Dajing, Jiangxi	114°33′	26°28′	3	37.50	1.625	1.2456	0.1403	0.2448	0.429
BG	C	11	Binguan, Jiangxi	114°33′	26°29′	3	37.50	1.375	1.2006	0.1198	0.1835	0.364
SJ	C	20	Shangjing, Jiangxi	114°34′	26°29′	5	62.50	2.000	1.1627	0.1219	0.2403	0.450
CQ	M	8	Jinyun Mount, Chongqing	106°13′	29°47′	3	37.50	1.375	1.0621	0.0533	0.1017	0.125
GD	C	20	Ruyuan, Guangdong	113°25′	24°47′	1	12.50	1.125	1.0131	0.0119	0.0248	0.100
LP	C	20	Lanping, Guangxi	109°18′	25°12′	5	62.50	1.750	1.2462	0.1463	0.2501	0.350
WM	C	13	Wuming, Guangxi	109°18′	25°12′	0	0.00	1.000	1.0000	0.0000	0.0000	0.231
Mean	M					2	25	1.30	1.08	0.06	0.19	0.23
	C					3.47	43.33	1.63	1.23	0.19	0.22	0.39
*p*‐values						.047	.047	.020	.014	.158	.703	.022

Abbreviations: C, central population; cpSSR, plastid simple sequence repeat; *h*, Nei's gene diversity; *H*_d_, haplotype diversity; *I*, Shannon's information index; M, marginal population; *N*_a_, observed number of alleles; *N*_e_: effective number of alleles; *N*_p_: number of polymorphic loci; PPB, percentage of polymorphic band.

### 2.3 DNA extraction

Genomic DNA was extracted using a modified cetyltrimethylammonium bromide (CTAB) procedure (Su et al., 2005). The DNA was quantified using a NanoDrop 2000c spectrophotometer (Thermo Fisher Scientific) and stored at −20°C at a final concentration of 50 ng/μl.

### 2.4 Primer selection and cpSSR analysis

We screened 29 plastid microsatellite primer pairs. The primers were designed by using the *Pinus thunbergii* chloroplast genome sequence or selected from published sources (Vendramin, Lelli, Rossi, & Morgante, 1996). SSRs were detected using MIcroSAtellite (MISA) (http://pgrc.ipk-gatersleben.de/misa/misa.html). The following parameter settings were applied: a motif size of 1–6 nucleotides, and at least 10 repeat units for mononucleotides and 5 for di‐, tri‐, tetra‐, penta‐, and hexanucleotides, respectively. Primer premier 5.0 (Clarke & Gorley, 2001) was used to design primers for SSRs. Eight primer pairs that produced clear bands were selected for further analysis (Table 2). Of these, the markers cpSSR11, cpSSR18, and cpSSR20 were described by Vendramin et al. (1996).

**Table 2:** Summary of the eight cpSSR loci used to study the population genetics among 22 populations of *Taxus wallichiana* var. *mairei*

Loci	Forward (F) and reverse (R) primers (5′–3′)	*N*_A_	*H*_T_	*H*_S_	*N*_a_	*N*_e_	*G*_ST_
cpSSR‐11	CACAAAAGGATTTTTTTTCAGTG CGACGTGAGTAAGAATGGTTG	3	0.0180	0.0178	3.0000	1.0239	0.0135
cpSSR‐18	AAAGCTTTTATTGCGGCC ATGGCAGTTCCAAAAAAGC	3	0.1246	0.0380	3.0000	1.1067	0.6953
cpSSR‐20	TAAGGGGACTAGAGCAGGCTA TTCGATATTGAACCTTGGACA	2	0.0337	0.0301	2.0000	1.0421	0.1075
cpSSR‐00	TTTGCAAGAAGGATGGCTAGA CGGCTCCTCCTTTCTTTACA	4	0.4626	0.3093	4.0000	1.6072	0.3314
cpSSR‐31	CGATTAGAACTTGAGTCGTTCAGG TCCTCGTCCAATCATTAATTCC	5	0.4013	0.2530	5.0000	1.6258	0.3696
cpSSR‐32	TTCATTAATCTTCAAACTGATTCG	3	0.0285	0.0280	3.0000	1.0300	0.0202
	TCCCAGAGGGATAAAATGAGG						
cpSSR‐33	CAAGCTGTTCAAGGCTATAATCTG CTCCATGGCAGAGAGAAAGG	4	0.3952	0.2413	5.0000	1.6102	0.3894
cpSSR‐34	TGTCCGGTCAGAACTTGTCA GCCCCAAACCAATAGACAGT	5	0.1881	0.1412	3.0000	1.2604	0.2492
Mean		3.625	0.2065	0.1323	3.5000	1.2883	0.3592

Abbreviations: cpSSR, plastid simple sequence repeat; *G*_ST_, population differentiation; *H*_S_, mean genetic diversity within populations; *H*_T_, total genetic diversity; *N*_A_, number of alleles; *N*_a_, observed number of alleles; *N*_e_, effective number of alleles.

PCR amplification was performed in a 25 μl final volume containing 1 × PCR buffer, 2.5 mM MgCl_2_, 0.2 mM of each dNTP, 0.2 μM of each primer, 1 U of *Taq* DNA polymerase, and 50 ng of template DNA. The PCR conditions were initial denaturation at 94°C for 5 min; followed by 25 cycles of 94°C for 60 s, 55°C for 70 s, and 72°C for 70 s; then a final extension step at 72°C for 8 min. Amplification products were separated using a 6% (w/v) denaturing polyacrylamide gel that contained 7 M urea and 1 × TBE. The gel was run at a constant voltage of 200 V for 120 min with a 20‐bp DNA ladder and silver‐stained (Tixier, Sourdille, Röder, Leroy, & Bernard, 1997).

### 2.5 Data analysis

Seven genetic parameters were calculated using FSTAT v. 2.9.3 (Goudet, 2001). These were the percentage of polymorphic bands (PPB), Shannon's information index (*I*), the observed number of alleles per locus (*N*_a_), the effective number of alleles per locus (*N*_e_), Nei's gene diversity index (*h*), the genetic differentiation coefficient (*G*_ST_), and gene flow (*N*_m_). *N*m was estimated as *N*_m_ = (1–*G*_ST_)/ 4*G*_ST_. The mean gene diversity within populations (*H*_S_) and total gene diversity (*H*_T_) were also estimated.

Analysis of molecular variance (AMOVA) was conducted to assess population differentiation within and among populations with 1,000 random permutation replicates using Arlequin software (ver. 3.0; Excoffier, Laval, & Schneider, 2005; Excoffier & Smouse, 1994). The values of pairwise fixation index (*F*_ST_) among populations were obtained by the same software. Because of the lack of recombination in the plastid genome, alleles identified at polymorphic loci were combined to generate the plastid haplotype for each individual (Wang, Guo, & Zhao, 2011). Using Arlequin, the haplotype diversity (*H*_d_) was calculated and the minimum spanning network determined. Mismatch distributions were used to investigate whether a population had undergone a sudden population expansion. The goodness of fit of spatial expansion was tested using the sum of squared deviations (SSD) and Harpending's raggedness index (HRag) (1994) with mismatch distributions based on 1,000 bootstrap replicates as implemented using Arlequin software. A dendrogram of 22 populations was inferred based on the unweighted pair group method with arithmetic average (UPGMA).

### 2.6 Comparison of genetic diversity between the central and marginal populations

The central and marginal *T. wallichiana* var. *mairei* populations were defined according to their geographical locations, based on our sampling and herbarium information (Table 1). We divided the range into two equal areas, peripheral and central (Channell & Lomolino, 2000). Two bands were established by collapsing the range boundaries with lines of longitude from both the western and eastern range limits until the areas occupied achieved the ratio of 1:2:1 (Yang et al., 2016; Figure 1).

Populations located at the western or eastern edge of the range were designated “peripheral/marginal populations” if their distances to the nearest edge were less than the distance to the center of the range, whereas populations situated in the center of the range were designated “central populations.” *Taxus wallichiana* var. *mairei* has a central range extending from Hunan, Hubei, to Jiangxi (Figure 1), and its peripheral populations are usually disjunct or isolated with a small population size. The genetic diversity and genetic differentiation of the central versus marginal populations were compared using *t* tests.

Besides longitude, we consider the distribution pattern of Taxus wallichiana var. mairei as well. The plant has a central range extending from Hunan, Hubei, to Jiangxi (Figure 1), and its peripheral populations are usually disjunct or isolated with a small population size.

### 2.7 Species distribution modeling

MAXENT software (ver. 3.3.3k; Phillips, Anderson, & Schapire, 2006) was used to predict the present and future distribution of *T. wallichiana* var. *mairei*. To further determine which climatic variables most affected distribution, we downloaded 19 bioclimatic variables from five different time periods: the present (1950‐2000), the Middle Holocene (MH; c. 6 kya), the last glacial maximum (LGM, c. 21 kya), the last interglacial (LIG, c. 130–114 kya), and the future (2070, average for 2061–2080) at a spatial resolution of 30 arcs from the WorldClim database (http://www.worldclim.org/; Hijmans, Cameron, Parra, Jones, & Jarvis, 2005). In addition to the 22 sample locations in this study, we also collected the distribution records of *T. wallichiana* var. *mairei* from the Chinese Virtual Herbarium (http://www.cvh.org.cn/). After removing duplicate records, a total of 65 *T. wallichiana* var. *mairei* records were used to generate the distribution model.

The Hadley Global Environment Model 2 (HadGEM2‐ES) with two climate scenarios (IPCC‐CMIP5 RCP 4.5/8.5) was selected to ensure predictive accuracy. The difference between these scenarios is the concentration of greenhouse gases. The RCP 8.5 scenario includes a higher concentration of emissions than the RCP 4.5 scenario. Model performance was evaluated using the area under the (receiver operating characteristic) curve (AUC) calculated by MAXENT. Values greater than 0.9 indicate good discrimination (Swets, 1988). The key parameters were set as follows: random test percentage = 25, maximum iterations = 500, and replicates = 15. Ultimately, the importance of environmental variables was determined by a jackknife test during modeling.

## 3 RESULTS

### 3.1 CpSSR variation, genetic diversity, and population structure

The eight plastid SSRs generated 176 bands from 339 individuals taken from the 22 populations surveyed. In total, 66 of these bands (37.50%) were polymorphic. The number of alleles per locus ranged from 3 to 5 with a mean of 3.63. The values of *H*_T_ and *H*_S_ ranged from 0.0180 to 0.4626 and 0.0178 to 0.3093, respectively, with a mean of 0.2065 and 0.1323 (Table 2). The observed number of alleles (*N*_a_) and the effective number of alleles (*N*_e_) ranged from 2 to 5 and from 1.0239 to 1.6258, respectively, with a mean of 3.5 and 1.2883 (Table 2).

At the population level, *N*_a_ ranged from 1.000 to 2.125 with a mean of 1.528, whereas *N*_e_ ranged from 1.000 to 1.416 with a mean of 1.181. Nei's gene diversity (*h*) at the species level was 0.145 and ranged from 0.000 to 0.976 (Table 1).

The TZ population had the greatest level of genetic diversity (*N*_a_ = 2.125, *N*_e_ = 1.380, *h* = 0.244, *I* = 0.411, PPB = 75%), followed by the SJ and LP populations (*N*_a_ = 2.000, *N*_e_ = 1.1627, *h* = 0.1219, *I* = 0.2403, PPB = 62.50%; *N*_a_ = 1.750, *N*_e_ = 1.2462, *h* = 0.1463, *I* = 0.2501, PPB = 62.50%), whereas no genetic diversity was detected in the QL, WM, and AH populations (Table 1).

The level of population differentiation was significant for all eight cpSSR loci with a mean *G*_ST_ value of 0.3592 (range: 0.0135–0.3894; Table 2). The standardized genetic differentiation data showed that all the pairwise fixation index (*F*_ST_) values among the populations were highly significant (*p* < .001), indicating that all the populations had differentiated significantly from the others. Comparing the TK and SJ populations produced the lowest *F*_ST_ value (0.00122), whereas comparing the QL and CQ populations produced the highest *F*_ST_ value (1.0000).

The hierarchical AMOVA revealed that 61.01% of the total variation in cpSSRs was attributable to differences among individuals within populations, 16.31% to differences among populations within regions, and 22.68% to differences among regions (Table 3). Genetic differences among and within populations were highly significant (*G*_ST_ = 0.3592, *p* < .01) (Table 2), which was consistent with the AMOVA analysis. The most substantial gene flow (*N*m = 408.00) occurred between the TK and SJ populations.

**Table 3:** Analysis of molecular variance (AMOVA) based on pairwise differences in cpSSRs for *Taxus wallichiana* var. *mairei*

Source of variance	*df*	SS	Variance components	Variance percentage (%)	*p*‐value
Among regions	8	72.038	0.18330Va	22.68	<.050
Among populations within regions	13	32.254	0.13185Vb	16.31	
Within populations	317	156.337	0.49318Vc	61.01	
Total	338	260.628	0.80834		

Abbreviations: cpSSRs, plastid simple sequence repeats; *df*, degrees of freedom; *p*‐value, significance tests after 1,000; SS, sum of squares.

**Figure 2:**
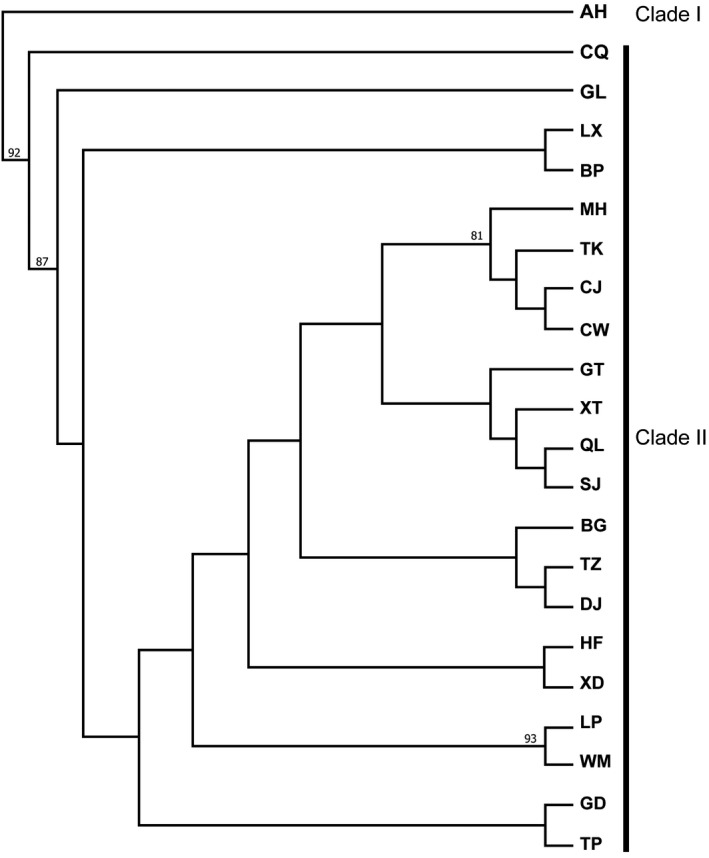
UPGMA dendrogram based on Nei's genetic distances among the 22 T*axus wallichiana* var. *mairei* populations. Abbreviation: UPGMA, unweighted pair group method with arithmetic average. Bootstrap values larger than 80 were shown above the branches

The UPGMA dendrogram separated the populations into two groups: Clade I and Clade II (Figure 2). The AH population formed an independent clade (Clade I) and the other populations clustered into a second (Clade II), which could be further subdivided into three subclades. The CQ and GL populations each formed separate subclades, whereas the remaining populations formed a third subclade. The CJ (Hubei), CW (Hubei), TK (Zhejiang), and MH (Fujian) populations were grouped together, although they originated from different provinces (Figure 2).

The cpSSRs produced 54 haplotypes with a mean *H*_d_ across populations of 0.363 (range: 0.100–0.600). Among the 54 haplotypes identified, 29 were private haplotypes. At the regional level, Hunan province had the largest number of private haplotypes (9; notably the TZ population had 5), whereas Guangdong had none. The haplotype richness per population and per region ranged from 1 (AH, QL, and CQ) to 12 (TZ) and 1 (Chongqing) to 26 (Hunan), respectively (Table S1). Hunan (52.00%) and Hubei (43.27%) provinces had relatively high haplotype diversity, whereas Anhui (9.09%) and Guangdong (10.00%) provinces had low values. Haplotype H3 appeared to be an ancient haplotype because it occurred across all populations except BP, AH, CQ, and QL. The minimum spanning network consisted of three main lineages. One contained individuals from all populations except AH and CQ (Figure S1, Table S2). Mismatch distribution analysis supported the spatial expansion model (SSD = 0.00429, *p* = .48200; HRag = 0.01413, *p* = .50600).

### 3.2 Genetic comparison of the central and peripheral populations

We divided the *T. wallichiana* var. *mairei* populations according to their geographical locations; there was one western marginal population, six eastern marginal populations, and 15 central populations (Figure 1, Table 1). There were no significant differences in genetic diversity between the central populations and the western marginal population (*p* > .05). However, higher levels of genetic diversity were found in the central populations compared to the eastern marginal populations (Table 1). Overall, with the exception of *h* and *I*, the genetic diversity parameters of the central populations were significantly higher than those of marginal populations (including *N*_p_, PPB, *N*_a_, *N*_e_, and *H*_d_; *p* = .014 − .047), although some central populations, such as GD, had low levels of genetic diversity (Table 1). Moreover, *F*_ST_ values for the central populations were significantly lower than those for marginal populations (*p* < .05).

### 3.3 Prediction of distribution ranges

**Figure 3:**
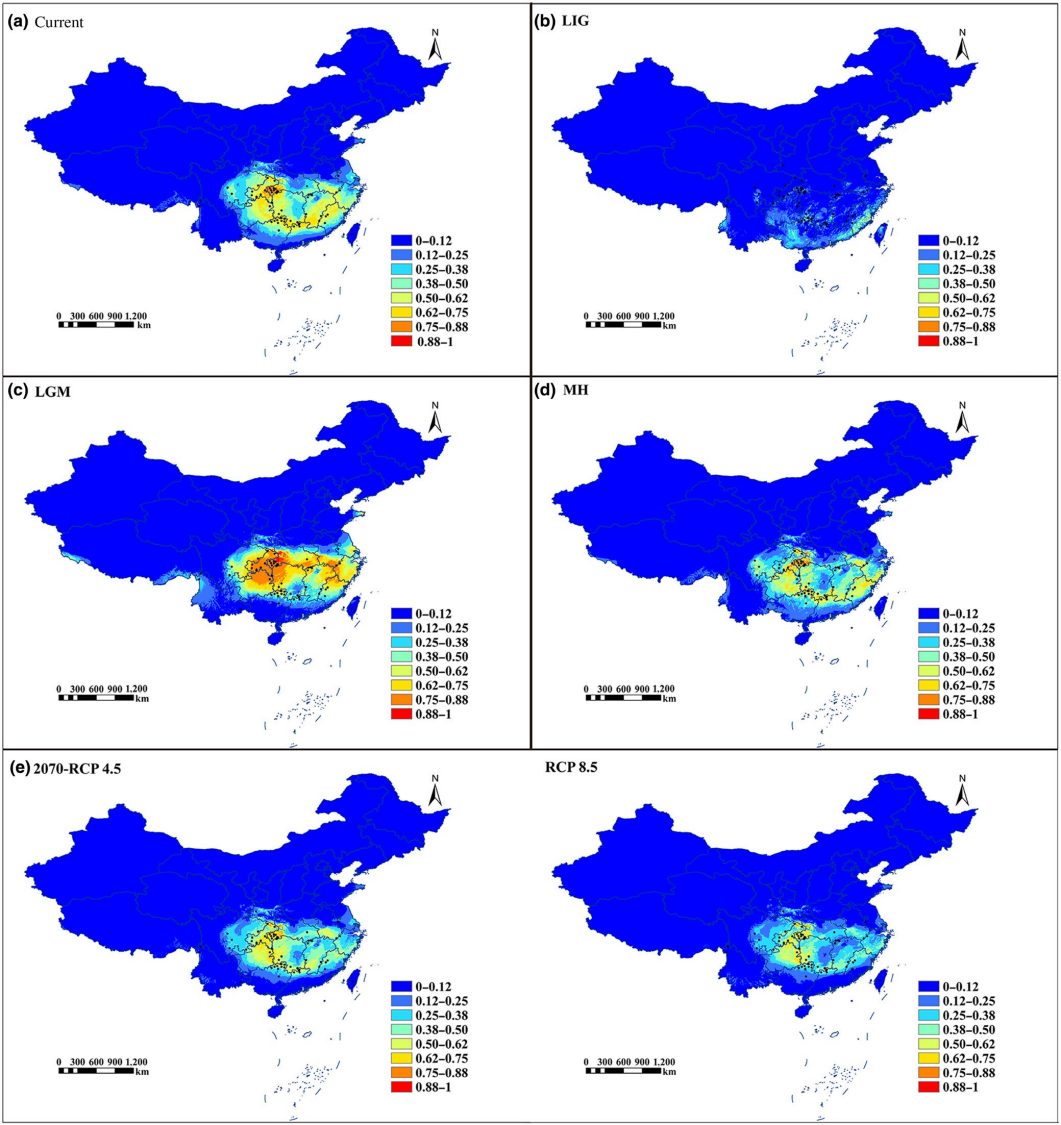
Predicted habitat for *Taxus wallichiana* var. *mairei*. (a) Present day. (b) Last interglacial (LIG, c. 120–140 kya). (c) Last glacial maximum (LGM, c. 21 kya). (d) Middle Holocene (MH, c. 6 kya). (e) Predicted in the future (2070, average for 2061–2080) based on RCP 4.5 and RCP 8.5. Color‐scale keys in each subfigure represent the habitat suitability

The MAXENT model for geographical distribution of *T. wallichiana* var. *mairei* performed well with an AUC value of 0.938. With the exception of eastern Guizhou province, the predicted distribution of *T. wallichiana* var. *mairei* under the present climate conditions generally corresponded with its current distribution in Jiangxi, Zhejiang, Guizhou, Guangxi, Fujian, and Guangdong provinces. These regions have climates that are suitable for *T. wallichiana* var. *mairei.* Palaeodistribution modeling suggested that *T. wallichiana* var. *mairei* had a more restricted range during the last interglacial (LIG) period but occupied a larger area, as a result of subsequent expansion, at the last glacial maximum (LGM) compared with its present distribution (Figure 3b,c). In addition, a range expansion occurred after the Middle Holocene (MH) (Figure 3a,d). Furthermore, compared with its present distribution, the future range suitable for *T. wallichiana* var. *mairei* is likely to decrease and shift northward under the RCP 4.5 and RCP 8.5 scenarios (Figure 3e). In particular, projections based on the RCP 8.5 scenario indicated that southern and southeastern regions, such as Jiangxi and Fujian provinces, would become significantly less favorable for *T. wallichiana* var. *mairei*. Additionally, in the future, *T. wallichiana* var. *mairei* distribution is likely to be sparser than at present.

**Figure 4:**
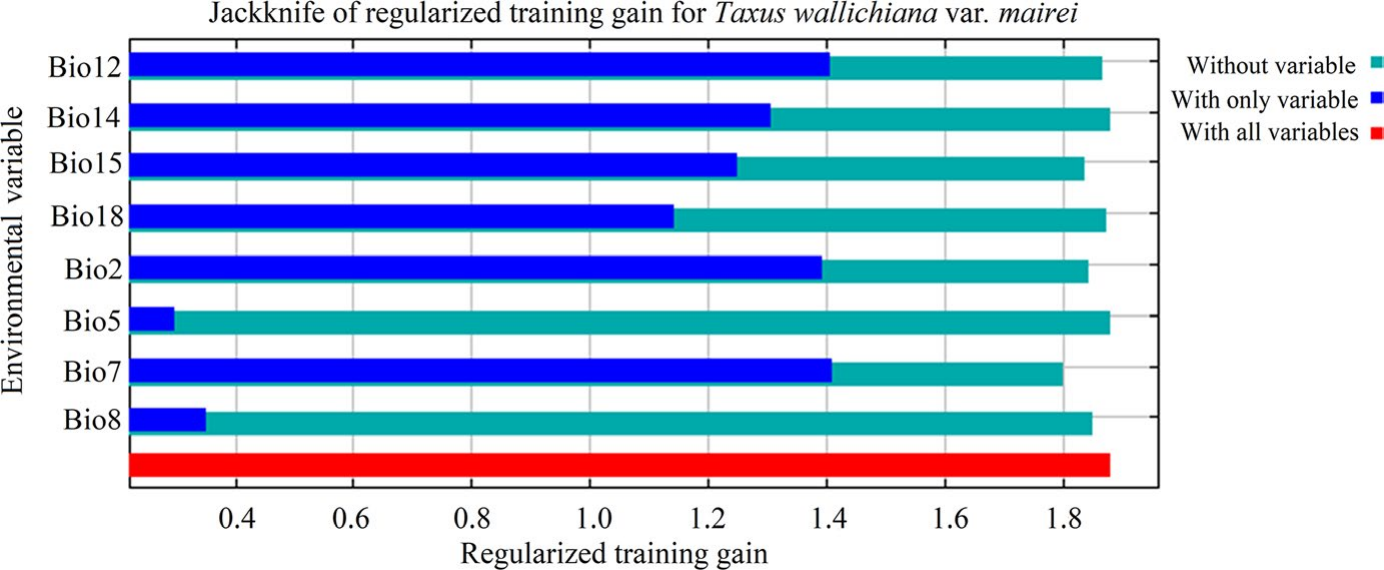
The Jackknife test for evaluating the relative importance of environmental variables for *Taxus wallichiana* var. *mairei*. Bio2: mean monthly temperature range; Bio5: maximum temperature during the warmest month; Bio7: annual temperature range (5–6); Bio8: mean temperature during the wettest quarter; Bio12: annual precipitation level; Bio14: precipitation levels during the driest month; Bio15: seasonal precipitation level (CV); Bio18: precipitation levels during the warmest quarter

**Figure 5:**
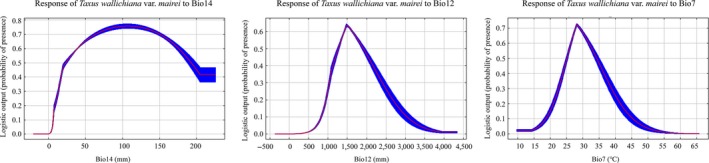
Response curves showing the relationship between environmental predictors and the probability of *Taxus wallichiana* var. *mairei* being present. Bio12: annual precipitation level; Bio14: precipitation levels during the driest month; Bio7: annual temperature range (5–6)

We investigated the most important climatic factors for determining the distribution of *T. wallichiana* var. *mairei.* Precipitation levels during the driest month (Bio14) contributed most, followed by annual precipitation level (Bio12), annual temperature range (Bio7), mean monthly temperature range (Bio 2), and mean temperature during the wettest quarter (Bio 8) (Figure 4, Table 4). The cumulative contribution of these five factors was 98%. As shown in Figure 5, the response curve indicated that *T. wallichiana* var. *mairei* was most likely to be present when the precipitation levels during the driest month (Bio 14) were 50–150 mm, the annual precipitation level (Bio 12) was 1,250–1,800 mm, and the annual temperature range (Bio7) was 25–35°C.

**Table 4:** Environmental variables examined in this study and their percentage contribution

Environmental variables	Unit	% contribution
Precipitation levels during driest month (Bio14)	mm	49.9
Annual precipitation level (Bio12)	mm	26.9
Annual temperature range (5–6) (Bio7)	°C	12.2
Mean monthly temperature range (Bio2)	°C	5.1
Mean temperature during wettest quarter (Bio8)	°C	3.9
Seasonal precipitation level (CV) (Bio15)	–	1.9
Maximum temperature during warmest month (Bio5)	°C	0
Precipitation levels during warmest quarter (Bio18)	mm	0

## 4 DISCUSSION

In this study, we used cpSSRs to investigate genetic diversity and population genetic structure in 22 populations of *T. wallichiana* var. *mairei.* Overall, *T. wallichiana* var. *mairei* had low levels of genetic variation although its PPB was slightly higher than other conifers (Table 5). The other genetic parameters such as the observed number of alleles and Shannon's information index also supported this conclusion. The total gene diversity of *T. wallichiana* var. *mairei* (*H*_T_ = 0.207) was lower than the mean cpSSR‐based gene diversity in 16 coniferous species (*H*_T_ = 0.801) (Petit et al., 2005). Similarly, the cpSSR haplotype diversity of *T. wallichiana* var. *mairei* (*H*_d_ = 0.363) was lower than the mean value (*H*_d_ = 0.670) detected in 170 plant species investigated by Petit et al. (2005). The low genetic diversity observed in *T. wallichiana* var. *mairei* may reflect a lack of accumulated nucleotide mutations in its plastid genome over the long evolutionary timescale. Provan et al. (1999) detected a low mutation rate at the cpSSR loci of the conifer *Pinus torreyana*, varying from 3.2 × 10^–5^ to 7.9 × 10^–5^.

**Table 5:** Some genetic parameters obtained from different conifer species based on cpSSR data

Species	*N*_A_	PPB (%)	*H*_T_	*H*_S_	*G*_ST_	Reference
*Pinus resinosa* Ait.	2.28		0.618	0.543	0.121	Echt, Deverno, Anzidei, and Vendramin (1998)
*Larix keampferi* Carr.	3.33		0.1662	0.1573	0.0537	Zhang, Bai, and Huang (2004)
*Pinus koraiensis* Sieb. et Zucc.			0.2149	0.1985	0.0762	Shao, Pei, and Zhang (2007)
*Pinus tabuliformis* Carr.		33.5	0.104	0.394	0.737	Yang (2009)
*Taiwania cryptomerioides* Hay.	4.4	23.91	0.4015	0.14	0.6512	Li (2014)
*Taxus wallichiana* var. *mairei* Lemee et Lév.	3.63	37.5	0.2065	0.1323	0.3592	This study

Abbreviations: cpSSR, plastid simple sequence repeat; *G*_ST_, population differentiation; *H*_S_, mean genetic diversity within populations; *H*_T_, total genetic diversity; *N*_A_, number of alleles; PPB, percentage of polymorphic bands.

We found that the genetic diversity of *T. wallichiana* var. *mairei* based on cpSSR markers was significantly lower than previously estimated using nuclear SSR and ISSR markers (Zhang & Zhou, 2013; Zhang et al., 2009). Similar observations have been reported for other species (Wang et al., 2011). One reason for this may be the lower mutation rate of cpSSRs compared with nuclear markers (Provan et al., 1999). Nonetheless, the small population sizes may have an effect as well. The QL, CQ, and AH populations have only 4, 8, and 11 individuals, respectively. Such small sizes could cause the reduction of genetic diversity due to genetic drift.

*Taxus wallichiana* var. *mairei* has relatively low genetic differentiation compared with other conifers, such as *Pinus tabuliformis*, *Pinus henryi*, and *Taiwania cryptomerioides* (Table 5). The UPGMA tree also showed that there was a considerable admixture among its populations (Figure 2). The cpSSR‐based AMOVA analysis indicated that most *T. wallichiana* var. *mairei* genetic differentiation occurred within populations, which is consistent with the nuclear SSR data but differs from the ISSR data (Zhang & Zhou, 2013; Zhang et al., 2009). Our cpSSR results provide information that complements the data from previous *T. wallichiana* var. *mairei* studies. The low genetic differentiation of *T. wallichiana* var. *mairei* could be linked to its biological characteristics*.* Long‐lived coniferous species have the capacity to create a buffer against genetic erosion (Templeton & Levin, 1979; Xu, Tremblay, Bergeron, Paul, & Chen, 2012). This involves using such traits as effective pollen or seed dispersal to elevate the level of gene flow and reduce population differentiation (Hamrick, Godt, & Sherman‐Broyles, 1992; Heuertz et al., 2004; Xu et al., 2012). However, we would not suggest that the low genetic differentiation revealed here implies there exists efficient gene flow between the extant populations of *T. wallichiana* var. *mairei*. Instead, it may reflect a time‐lag effect of historical influences, for example, the plant was distributed more contiguously before the Quaternary glaciation (Yue, Chen, Guo, & Wang, 2011).

It is generally believed that refugial populations have high levels of genetic diversity and a large number of private haplotypes (Bhagwat & Willis, 2008; Comes & Kadereit, 1998; Stewart, Lister, Barnes, & Dalén, 2010). The large number of cpSSR private haplotypes and greater genetic diversity of *T. wallichiana* var. *mairei* detected in the populations from Hunan and Hubei provinces suggest that these regions probably serve as major refugia. Gao et al. (2007) previously argued that central China, including Hunan and Hubei, provided important refugia for *T. wallichiana* populations with ancient haplotypes, and this suggestion is supported by our MAXENT results. A spatial expansion from a stable population was also detected by mismatch analysis. In addition, our MAXENT analysis showed that *T. wallichiana* var. *mairei* had experienced two expansions into potentially suitable habitats: one between the LIG and the LGM and the other between the MH and the present. Moreover, the star‐like shape of the minimum spanning network further suggests that *T. wallichiana* var. *mairei* experienced a relatively recent range expansion (Figure S1). As a temperate plant, *T. wallichiana* var. *mairei* may not be directly affected by periods of glaciation. Its refugia in central China represent distribution centers from which peripheral populations may colonize previously glaciated regions (Hampe & Petit, 2005; Hewitt, 1999; Lesica & Allendorf, 1995).

We found that genetic diversity in *T. wallichiana* var. *mairei* decreased from the core to the peripheral populations for all genetic parameters except two. We also found that levels of genetic differentiation were greater in the peripheral populations. These findings from cpSSR data support the predictions of the CMH. After reviewing 134 nuclear marker‐based studies, Eckert et al. (2008) concluded that 64.2% of them detected reduced genetic diversity and 70.2% found increased genetic differentiation between peripheral and central populations. However, plastid genome data were not included. The central regions, which included the proposed refugia of Hunan and Hubei provinces, represented the distribution core and were defined as the geographical center (Micheletti & Storfer, 2015). In particular, the TZ population was identified as the most centrally located population due to its high genetic diversity and large number of private alleles in comparison with other populations (Figure 1). Central populations may have a significant influence on the migration of plant populations, and the shape of the minimum spanning network suggests an asymmetric migration pattern (Figure S1). The causes of reduced cpSSR diversity and increased differentiation from the central to the peripheral *T. wallichiana* var. *mairei* populations may include increased isolation, reduced effective population size and a corresponding increase in genetic drift, and historical demographic events (Christiansen & Reyer, 2011; Pfeifer et al., 2009). Importantly, strong adaptation to local conditions may also have a critical effect (Cahill & Levinton, 2016).

The factors that govern the range limits of *T. wallichiana* var. *mairei* should be investigated as a priority, especially in the context of global warming (Trumbo et al., 2016; Ursenbacher et al., 2015). Ranges are not simply limited by physiological tolerances or obvious dispersal barriers. Instead, they may be defined by the inability of peripheral populations to adapt to the prevailing conditions beyond their current range (Micheletti & Storfer, 2015). The CMH predicts that peripheral populations are prevented from expanding beyond their current range because they act as demographic sinks with decreased fitness. Therefore, a plan for conserving the peripheral populations of *T. wallichiana* var. *mairei* should be devised urgently.

Our MAXENT model generated an accurate prediction with good AUC values. The results obtained for the time period we investigated suggested that *T. wallichiana* var. *mairei* probably underwent cycles of expansion and contraction, and its genetic and phylogeographic structure were hugely affected by Quaternary climatic fluctuations. The predicted distribution of *T. wallichiana* var. *mairei* for the present time corresponds well to its actual distribution. In general, precipitation and temperature strongly determine the geographical distribution of the species. We found that precipitation levels during the driest month (Bio14), annual precipitation level (Bio12), and annual temperature range (Bio7) were the most important factors, indicating that *T. wallichiana* var. *mairei* prefers moist environments, and rainfall patterns are decisive in determining its distribution (Wu & Wen, 2017; Zhang et al., 2009). The mean monthly temperature range (Bio 2) and the mean temperature of the wettest quarter (Bio 8) are also important factors. Future projections reveal that a greater reduction in areas suitable for *T. wallichiana* var. *mairei* habitation would occur under the RCP 8.5 compared with the RCP 4.5 scenario, which suggests that this species is sensitive to a high concentration of greenhouse gases. As the future temperature is predicted to increase 2.3–2.7°C by 2070 (Solomon et al., 2007), we speculate that global warming will have a significant impact on the distribution of *T. wallichiana* var. *mairei.* A substantial loss of area suitable for habitation will occur and the plant will shift northward. Our data may provide a starting point from which to devise a conservation strategy for *T. wallichiana* var. *mairei* in the face of climate change.

## 5 CONCLUSIONS

We examined the *Taxus wallichiana* var. *mairei* plastid genome and found it had low levels of genetic variation and genetic differentiation. The cpSSR‐based genetic data complement previously published results based on nuclear SSRs and ISSRs. Our results support the CMH, which provides a theoretical basis for the conservation and management of peripheral populations. The suitable range of *T. wallichiana* var. *mairei* was predicted to decrease and shift northward in response to global warming. In addition, the most important climatic factors that limit the distribution of *T. wallichiana* var. *mairei* were identified. These included the levels of precipitation during the driest month (Bio14), annual precipitation level (Bio12), and annual temperature range (Bio7). It would be interesting in a future study to further compare phenotypic plasticity and epigenetic variation between the central and marginal populations of *T. wallichiana* var. *mairei* in the climate change context (Anderson et al., 2012).

## CONFLICT OF INTEREST

The authors declare there are no competing interests.

Consent for publication: Not applicable.

## AUTHOR CONTRIBUTIONS

TW designed the research and was involved in writing the manuscript; LL and ZW conducted data analysis and were involved in writing the manuscript and checking English grammar; LH performed the cpSSR experiment and conducted data analysis; and YS helped to supervise the research and was involved in writing the manuscript. All authors read and approved the final version of the manuscript.

## ETHICS APPROVAL AND CONSENT TO PARTICIPATE

The acquisition of plant material used in this study complies with institutional, national, and international guidelines. No specific permits were required for the described field studies. No specific permissions were required for the locations/activities described in this study.

## Data Availability

The datasets used for this study are available through Dryad at the time of publication (https://doi.org/10.5061/dryad.c8c79h7).
