# Supplementary material for: Chloroplast population genetics reveals low levels of genetic variation and conformation to the central–marginal hypothesis in Taxus wallichiana var. mairei, an endangered conifer endemic to China
Source: Ecol Evol. 2019 Sep 27;9(20):11944–56. doi: 10.1002/ece3.5703 (PMC6822043; doi:10.1002/ece3.5703)
Supplement: Supplementary file 1 [file ECE3-9-11944-s001.docx]

**Supporting Information**

**Table S1** Haplotypes of *Taxus wallichiana* var*. mairei* within 9 regions

| **Regions** | **Number of haplotypes** | **Number of samples** | **Number of private haplotypes** | **Haplotype diversity** |
| --- | --- | --- | --- | --- |
| Hunan | 26 | 50 | 9 | 0.5200 |
| Hubei | 45 | 104 | 7 | 0.4327 |
| Fujian | 12 | 48 | 2 | 0.2500 |
| Anhui | 1 | 11 | 1 | 0.0909 |
| Zhejiang | 7 | 20 | 1 | 0.3500 |
| Jiangxi | 19 | 45 | 3 | 0.4222 |
| Chongqing | 1 | 8 | 1 | 0.1250 |
| Guangdong | 2 | 20 | 0 | 0.1000 |
| Guangxi | 10 | 33 | 5 | 0.3030 |
| Total | 123 | 339 | 29 | 0.3628 |

**Table S2** Code number of minimum spanning network of *Taxus wallichiana* var*. mairei*

| **AH143** | **178** | **GD18** | **655** | **227** | **83** | **102** | **265** | **56** |
| --- | --- | --- | --- | --- | --- | --- | --- | --- |
| AH143 | 179 | GD19 | 656 | 228 | 83 | 196 | 573 | **129** |
| AH144 | 180 | GD20 | 657 | 232 | 41 | 104 | 585 | 129 |
| AH145 | 181 | LP18 | 658 | 246 | 42 | 647 | **542** | 143 |
| AH146 | 182 | LP24 | 604 | 247 | 77 | 197 | 542 | 144 |
| AH147 | 184 | LP25 | 605 | 248 | **624** | 649 | 538 | **190** |
| AH148 | 185 | LP27 | 606 | 249 | 624 | 234 | 609 | 190 |
| AH149 | 186 | LP28 | 608 | 252 | 611 | 235 | 574 | 191 |
| AH150 | 187 | LP30 | 610 | 253 | **LP10** | 237 | **535** | 607 |
| AH151 | 188 | LP31 | 64 | 256 | LP10 | 239 | 535 | 622 |
| AH152 | 189 | WM2 | 67 | 257 | 581 | 242 | 225 | **536** |
| AH153 | 163 | WM3 | 69 | 259 | 530 | 193 | 226 | 536 |
| **CQ1** | 164 | WM4 | 71 | 261 | 558 | 224 | 601 | 546 |
| CQ1 | 165 | WM5 | 79 | 268 | LP4 | 194 | 602 | **554** |
| CQ2 | 166 | WM6 | 575 | 200 | LP5 | 659 | 593 | 554 |
| CQ3 | 167 | WM8 | 577 | 201 | LP7 | 603 | 571 | 552 |
| CQ4 | 168 | WM9 | 579 | 202 | 240 | 198 | 207 | 555 |
| CQ5 | 169 | WM10 | 580 | 203 | 241 | 215 | 43 | 556 |
| CQ6 | 170 | WM11 | 586 | 204 | LP1 | **150** | **118** | LP15 |
| CQ7 | 171 | WM12 | 589 | 211 | LP2 | 150 | 118 | **587** |
| CQ8 | 172 | WM13 | 592 | 212 | LP3 | 142 | 199 | 587 |
| **GD23** | 173 | WM14 | 595 | 213 | **597** | 128 | 99 | 121 |
| GD23 | GD1 | 621 | 534 | 214 | 597 | **138** | 100 | **264** |
| 40 | GD2 | 623 | 548 | 216 | 31 | 138 | **641** | 264 |
| 44 | GD3 | 625 | 550 | 217 | 220 | 130 | 641 | 206 |
| 45 | GD4 | 627 | 560 | **155** | 223 | 140 | 532 | **114** |
| 46 | GD5 | 628 | 562 | 155 | 229 | 151 | 269 | 114 |
| 50 | GD6 | 630 | 32 | 126 | **195** | **LP22** | **125** | 208 |
| **157** | GD7 | 631 | 33 | 127 | 195 | LP22 | 125 | 209 |
| 157 | GD9 | 632 | 34 | 131 | 633 | LP19 | 123 | WM7 |
| 158 | 167 | 643 | 103 | 132 | 642 | **540** | **243** | **134** |
| 159 | 167 | 646 | 116 | 135 | 60 | 540 | 243 | 134 |
| 160 | 166 | 648 | 117 | 136 | 645 | 545 | 660 | 47 |
| 161 | 165 | 650 | 233 | 137 | 35 | 583 | 258 |  |
| 174 | 164 | 651 | 236 | 36 | 39 | 53 | 629 |  |
| 175 | 164 | 652 | 219 | 38 | 96 | 599 | 90 |  |
| 176 | GD16 | 653 | 221 | 152 | 97 | 230 | **156** |  |
| 177 | GD17 | 654 | 222 | 153 | 101 | 254 | 156 |  |


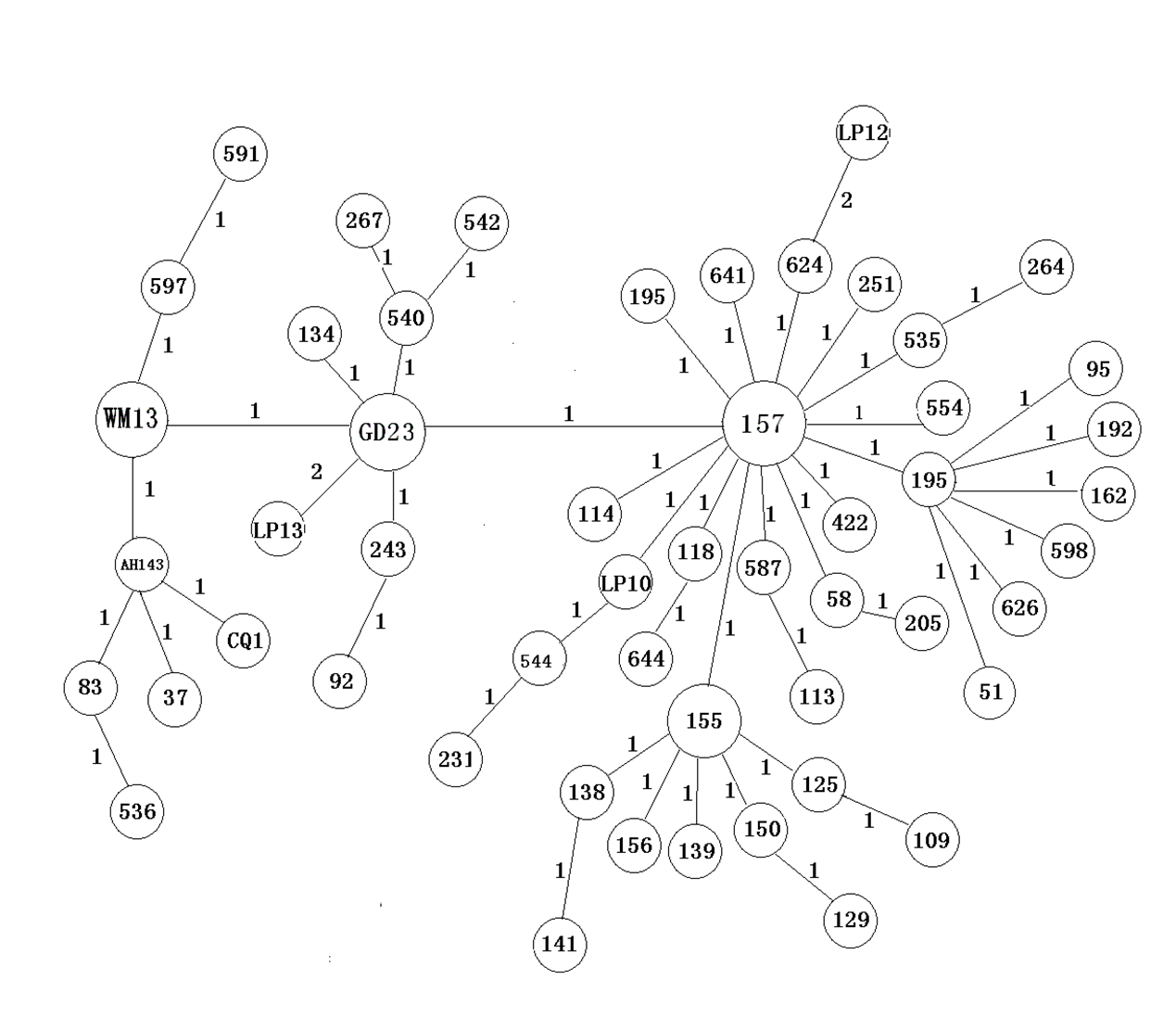


**Figure S1** Minimum spanning network of *Taxus wallichiana* var. *mairei* based on cpSSR data set. Numbers indicated next to the connecting lines represent the number of mutational steps. Numbers and letters within circles represent the haplotype codes.
